# RNAi pathway participates in chromosome segregation in mammalian cells

**DOI:** 10.1038/celldisc.2015.29

**Published:** 2015-10-20

**Authors:** Chuan Huang, Xiaolin Wang, Xu Liu, Shuhuan Cao, Ge Shan

**Affiliations:** 1 School of Life Sciences, CAS Key Laboratory of Brain Function and Disease, University of Science and Technology of China, Hefei, China

**Keywords:** RNAi pathway, chromosome segregation, CENPC1, AGO2, Dicer, α-satellite RNA, ASAT siRNA

## Abstract

The RNAi machinery is a mighty regulator in a myriad of life events. Despite lines of evidence that small RNAs and components of the RNAi pathway may be associated with structure and behavior of mitotic chromosomes in diverse organisms, a direct role of the RNAi pathway in mammalian mitotic chromosome segregation remains elusive. Here we report that Dicer and AGO2, two central components of the mammalian RNAi pathway, participate in the chromosome segregation. Knockdown of Dicer or AGO2 results in a higher incidence of chromosome lagging, and this effect is independent from microRNAs as examined with DGCR8 knockout cells. Further investigation has revealed that α-satellite RNA, a noncoding RNA derived from centromeric repeat region, is managed by AGO2 under the guidance of endogenous small interference RNAs (ASAT siRNAs) generated by Dicer. Furthermore, the slicer activity of AGO2 is essential for the chromosome segregation. Level and distribution of chromosome-associated α-satellite RNA have crucial regulatory effect on the localization of centromeric proteins such as centromere protein C1 (CENPC1). With these results, we also provide a paradigm in which the RNAi pathway participates in vital cellular events through the maintenance of level and distribution of noncoding RNAs in cells.

## Introduction

The centromere is a special region of chromosome needed for successful assembly of kinetochore and chromosome attachment to spindle [1–4]. Centromeres consist of long stretch of species-specific DNA repeats in most eukaryotes, and it has been known for more than a decade that some transcripts are derived from centromeric repeat regions in multiple eukaryotic organisms investigated [2, 3]. Although the organization of centromere DNA as repeats is largely conserved in eukaryotes, the repeat sequences, transcripts from the repeats and the molecular mechanisms underneath the centromere/kinetochore assembly are not that conserved among different species.

The core centromere of mammalian chromosome is the region for the assembly of kinetochore, and it consists of α-satellite repeats in human and minor satellite repeats in mice. Centromere protein B (CENP-B) is a highly conserved centromeric DNA-binding protein recognizing and binding to a 17-bp sequence (CENP-B box) in the centromeric α-satellite DNA [5, 6]. In vertebrates and mammals, studies have demonstrated that transcripts from the centromeric core associate with centromeric core proteins to serve as integral components of the centromere and kinetochore [2, 3, 7–10]. Several studies have shown that RNA polymerase II-dependent α-satellite RNA is an integral part of centromere and exerts an important function in the kinetochore assembly [7–9]. Centromeric α-satellite RNA associates directly with centromere protein C1 (CENPC1) and facilitates nucleoprotein assembly at the mitotic centromere [7, 11–13]. CENPC1 has both RNA-binding and DNA-binding capacity [7, 8]. This protein is essential for the integrity and localization of kinetochore, and loss of CENPC1 induces chromosome lagging [14, 15]. In mice, centromeric noncoding RNA (ncRNAs) of 120 nucleotides (nt) associate with centromeres and accumulate under stress or differentiation conditions [16]. Components of kinetochore are recruited to minor satellite repeat RNA by CENP-A [17, 18]. In mouse embryonic stem cells, loss of Dicer reduces the level of small double-stranded RNA from centromeric repeats [19]. In a chicken–human hybrid cell line, loss of Dicer results in a higher level of transcripts from α-satellite sequences [20].

The RNA interference (RNAi)/microRNA (miRNA) pathway in mammalian cells is mediated by a cascade of enzymatic reactions [21–23]. First, the large primary miRNA transcripts are processed by a complex of DGCR8 and Drosha into miRNA precursors of ~70 nt with a hairpin structure in the nucleus. miRNA precursors are then transported into the cytoplasm and get cut by Dicer to give rise to mature miRNAs. Endogenous or exogenous double-stranded RNA or short hairpin RNA can also be processed by Dicer to form small interference RNA (siRNA). Both miRNA and siRNA can be loaded into RNA-induced silencing complex (RISC) with its core component as AGO2. Inside the siRNA-programmed RISC, the siRNA triggers the degradation of targeted complementary RNA with the slicer activity of AGO2. Whereas in the miRISC (miRNA-programmed RISC), miRNA generally suppresses the translation or promotes the degradation of the target mRNA by incomplete complementation.

Previous results showed that small RNAs and components of the RNAi pathway might be associated with mitotic chromosome segregation [1–3, 24, 25], although the mechanism of how the RNAi machinery participates into this event remains elusive. In this study, we set out to investigate whether the RNAi pathway participates in mitotic chromosome segregation directly. We found that knockdown of Dicer or AGO2 resulted in chromosome lagging. With a DGCR8 knockout cell line, we demonstrated that this effect was largely independent from miRNAs. The RNAi pathway under the guidance of endogenous siRNAs kept the level of α-satellite RNA balanced for precise centromere/kinetochore formation. In addition, the slicer activity of AGO2 was essential, indicating further that a siRNA rather than a miRNA pathway was involved. Our data suggest that AGO2 guided by endogenous siRNAs generated by Dicer controls the level and localization of chromosomal α-satellite RNA to direct the deposition of important centromeric factors such as CENPC1 for mitotic chromosome segregation in mammalian cells.

## Results

### Dicer or AGO2 knockdown leads to defect in mammalian chromosome segregation

**Figure 1:**
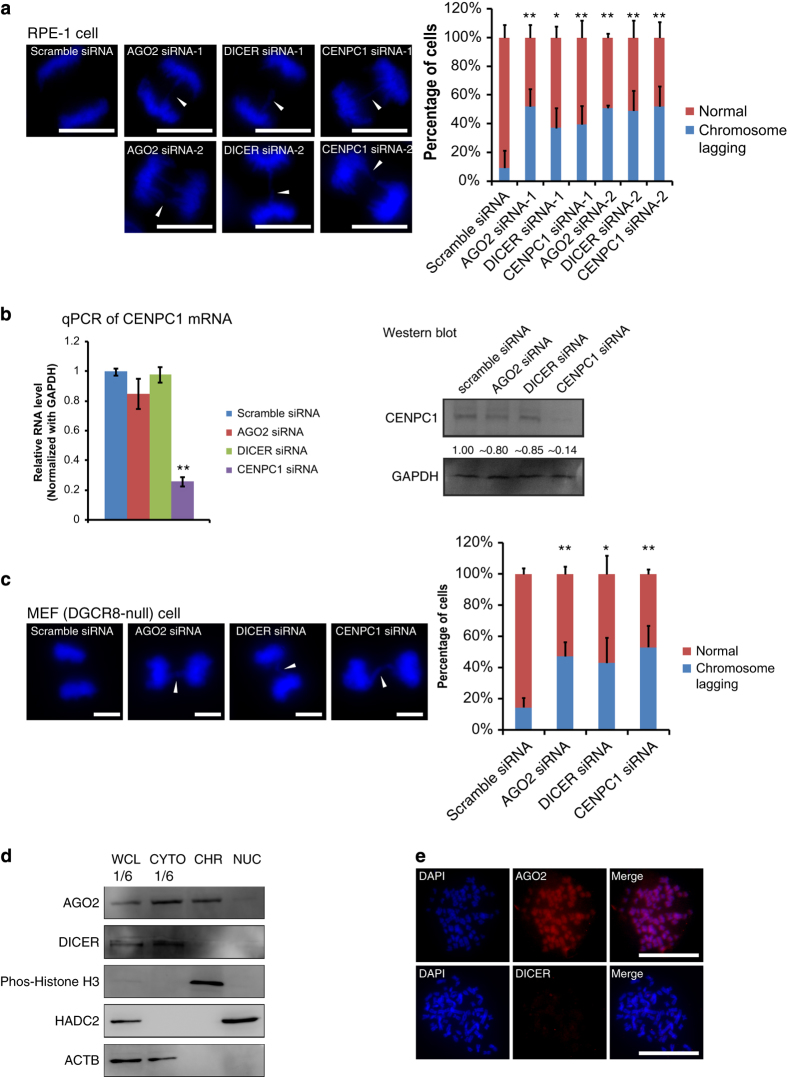
AGO2 and Dicer are required for proper chromosome segregation. (**a**) Knocking down of AGO2, Dicer and CENPC1 with two sets of siRNAs in human RPE-1 cells. Representative images of chromosome lagging during anaphase were shown (arrowheads) and the statistics of the results were shown on the right (*n*>60 cells per experiment). (**b**) Quantitative PCR (left) and western blot assay (right) depicted that CENPC1 mRNA and protein levels did not change significantly in AGO2 and Dicer knockdown HeLa cells. (**c**) Representative images of chromosome lagging (arrowheads) during anaphase upon knocking down of AGO2 and Dicer, respectively, with siRNAs in mouse DGCR8 null mouse embryonic fibroblast (MEF) cells. The statistical results were shown on the right (*n*>60 cells per experiment). (**d**) Western blot assay with whole-cell lyses (WCL), cytoplasm (CYTO), chromosome (CHR) and nuclei (NUC) samples showed that AGO2 rather than Dicer was on metaphase chromosomes of HeLa cells. Each sample contains the corresponding fraction from the same number of cells. Only 1/6 of the WCL and CYTO were loaded to avoid saturation of the corresponding bands. Phos-histone H3 (chromosome marker), HDAC2 (nucleus marker) and ACTB (Beta-Actin, cytoplasm marker). (**e**) Immunostaining of AGO2 and Dicer on metaphase chromosomes of RPE-1 cells showed that AGO2 but not Dicer was detected. Scale bar represents 10 μm. *P-value <0.05; **P-value <0.01. *P*-values were determined with two-tailed Student’s *t*-test. All data were from three repeats. Error bars represent s.d.

We started to investigate roles of the RNAi pathway in chromosome segregation by knocking down Dicer or AGO2 with siRNAs. Dicer or AGO2 knockdown resulted in chromosome lagging in mitotic human RPE-1 and HeLa cells (Figure 1a and Supplementary Figure S1A–E). As a comparison and also a positive control, knocking down of CENPC1 resulted in higher incidence of chromosome lagging (Figure 1a and Supplementary Figure S1B), and this phenomenon was consistent with early studies about roles of CENPC1 [14, 15, 26]. Furthermore, the mRNA and protein levels of CENPC1 were not significantly changed when knocking down the Dicer or AGO2, respectively (Figure 1b).

Functions of Dicer and AGO2 are largely associated with two classes of small RNA, miRNA and siRNA [23]. To see whether miRNAs are important, we next carried out AGO2 and Dicer knockdown experiments in DGCR8 knockout MEF cells [27–30]. It is well known that these cells lack functional miRNAs, as DGCR8 is a critical component of microprocessor for the production of pre-miRNA from pri-miRNA [27–30] (Supplementary Figure S1F). In DGCR8 knockout cells, AGO2 or Dicer knockdown caused a higher incidence of chromosome lagging (Figure 1c and Supplementary Figure S1G).

Interestingly, knocking down the other Argonaute family members including AGO1, AGO3 and AGO4 did not induce chromosome lagging (Supplementary Figure S2). It seems that Dicer, as well as AGO2 rather than the other Argonautes, possesses unique function in mitotic chromosome segregation.

Taken together, our results demonstrated that AGO2 and Dicer acted in mammalian mitotic chromosome segregation. Their roles in this cellular event may be independent from miRNAs.

### AGO2 but not dicer is a chromosomal protein

To investigate further roles of Dicer and AGO2 on mitotic chromosome segregation, we isolated human mitotic metaphase chromosomes with gradient centrifugation (Supplementary Figure S3A) [31]. Results from flow cytometry and immunostaining of chromosome marker (Phos-histone H3) indicated that the isolated chromosomes were of high purity (Supplementary Figure S3B and C). Chromosomes isolated with gradient centrifugation were mostly used for western blot analyses and RNA isolation in this research. Western blot analysis with chromosome samples showed that AGO2 rather than Dicer was a chromosomal protein (Figure 1d). For further confirmation, we applied an immunostaining assay with AGO2 or Dicer antibodies on mitotic chromosomes. Metaphase chromosome spreads were prepared by dropping for immunostaining assay. This method was used for all chromosomal immunofluorescent (IF) staining and RNA fluorescence *in situ *hybridization (FISH) in this study, unless specified. The immunostaining assay verified that there was AGO2 but almost no Dicer on metaphase mitotic chromosomes (Figure 1e and Supplementary Figure S3B and C). A previous proteomics survey of chicken metaphase chromosomes has shown that AGO2 (ranked 2 356th of 4 030 proteins) rather than Dicer (ranked 3 879th of 4 030 proteins) is a chromosomal protein [32]. In addition, AGO1, AGO3 and AGO4 did not locate on metaphase chromosomes (Supplementary Figure S3D). Our western blots and immunostaining also showed limited signals of AGO2 in the interphase nucleus (Figure 1d and Supplementary Figure S3E), and the data indicate that AGO2 might ‘flood into’ the nucleus presumably because of the increase in permeability or the dissembling of the nuclear envelope in the prometaphase (Supplementary Figure S3E).

### α-satellite RNAs are chromosomal ncRNAs and regulate mammalian chromosome segregation

**Figure 2:**
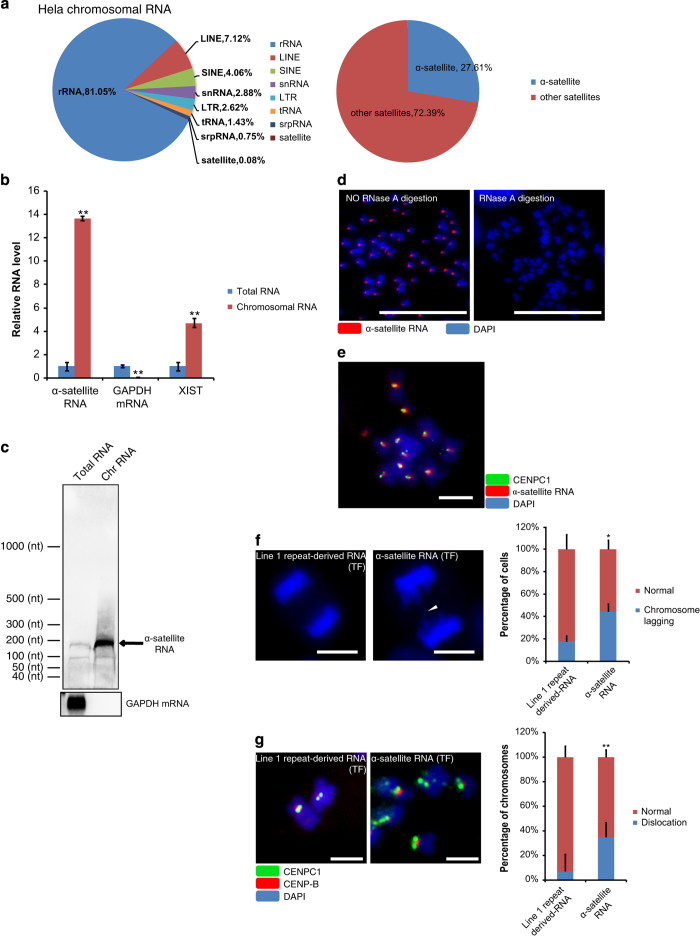
α-satellite RNAs are chromosomal ncRNAs and regulate mammalian chromosome segregation. (**a**) Composition of different kinds of repeat-derived chromosomal RNAs from HeLa cells. (**b**) Quantitative PCR to assess enrichment of α-satellite RNAs using chromosomal and total RNA from HeLa cells. Equal amount of RNA was used for each assay. GAPDH mRNA (a cytoplasmic RNA) and XIST RNA (a known chromosomal RNA) were examined for comparison. (**c**) Northern blot of chromosomal RNA and total RNA from HeLa cells for α-satellite RNAs. Equal amount of RNA was used for each assay. GAPDH mRNA was examined as a reference. (**d**) RNA-FISH assay for α-satellite RNA on RPE-1 metaphase chromosomes with or without RNase A treatment. The slides treated with RNase A did not have any signal, whereas the slides without RNase A treatment have FISH signals, which confirms the specificity of FISH signals for α-satellite RNAs but not the corresponding DNA loci. (**e**) RNA-FISH assay for α-satellite RNA combined with immunostaining of CENPC1 showed colocalization of α-satellite RNA with CENPC1 on RPE-1 metaphase chromosomes. (**f**) Effect of the transfection (TF) of *in vitro* synthesized α-satellite RNA, with Line 1 repeat-derived RNA as the negative control in human RPE-1 cells. Representative images of chromosome lagging (arrowheads) during anaphase were shown and the statistics of the results were shown on the right (*n*>60 cells per experiment). (**g**) Immunostaining of CENPC1 and CENP-B on RPE-1 metaphase chromosomes from cells transfected with α-satellite RNA or Line 1 repeat-derived RNA, respectively. ‘Dislocation’ refers both unequal loading of CENPC1 to centromeres and mislocation of CENPC1 to the chromosome arm. Quantification of CENPC1 dislocation was shown with bar figure (*n*>300 chromosomes per experiment). Scale bar represents 10 μm for **d** and **f**, 1 μm for **e** and **g**. *P-value <0.05; **P-value <0.01. *P*-values were determined with two-tailed Student’s *t*-test. All data were from three repeats. Error bars represent s.d.

Multiple lines of evidence have shown that ncRNAs are transcribed from centromeric repeats [2, 3]. We performed RNA sequencing with HeLa sample of metaphase chromosomal RNAs (Supplementary Figure S3A). Bioinformatic analysis of sequencing data for reads originated from genomic repeats showed that α-satellite RNAs, ncRNAs derived from centromeric repeats, were present on human mitotic chromosomes along with some other repeat-derived ncRNAs (Figure 2a). We confirmed the relative enrichment of α-satellite RNA in chromosomal RNA as against the total RNA with real-time polymerase chain reaction (PCR; Figure 2b). Northern blots showed that α-satellite RNAs with the length ~171 nt (the length of one unit of α-satellite repeat) were enriched on metaphase chromosomes (Figure 2c). Previous work has shown the presence of ~171 nt α-satellite RNAs along with transcripts of various lengths in an array of human cells [33, 34], and our northern blot with chromosomal RNAs demonstrated that the ~171-nt RNA is the major chromosomal α-satellite RNA. To gain insight into the subchromosomal distribution of α-satellite RNAs, we performed RNA-FISH (with or without pre-treatment of RNase A). Positive RNA-FISH signals were observed exclusively on the centromeric region of metaphase chromosomes (Figure 2d). Furthermore, co-staining of α-satellite RNA and CENPC1 protein showed that α-satellite RNA colocalized with CENPC1 (Figure 2e). Next, we found that transfection of *in vitro* synthesized α-satellite RNA (with Line 1 repeat-derived RNA as a negative control) into nuclei of human HeLa cells resulted in higher incidence of lagging chromosomes during mitosis (Figure 2f and Supplementary Figure S4A and B). This result was consistent with previous report about chromosome missegregation in murine cells with overexpressed centromeric RNA ^16^. Immunostaining of CENPC1 and CENP-B (a centromere marker [5, 6]) revealed that α-satellite RNA added *in trans* resulted in CENPC1 dislocation on chromosomal loci without CENP-B colocalization (Figure 2g).

Taken together, these results showed that α-satellite RNAs were component of metaphase chromosomes. Too many of these RNAs would be deteriorative to the formation and function of centromere/kinetochore as shown by CENPC1 dislocation and further chromosome lagging in mammalian cells.

### AGO2 and Dicer manage levels of α-satellite RNA and further localization of CENPC1

**Figure 3:**
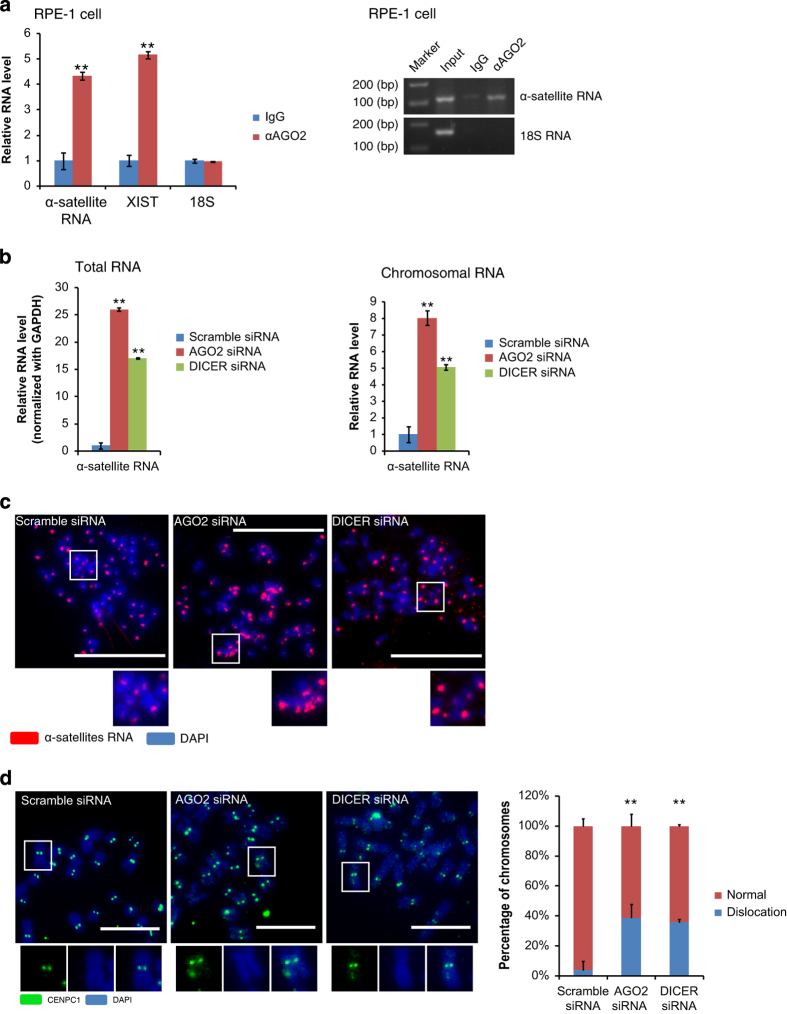
AGO2 and Dicer manage levels of α-satellite RNA and localization of CENPC1. (**a**) AGO2 RIP assay with RPE-1 chromosome samples followed by quantitative PCR and semi-quantitative PCR for α-satellite RNA. 18 S rRNA (negative control) and XIST RNA (a known AGO2-binding target on the chromosome) were also examined. (**b**) Quantitative PCR of α-satellite RNA with RPE-1 total RNA and chromosomal RNA samples upon AGO2 and Dicer knockdown, respectively. (**c**) RNA-FISH assay to detect α-satellite RNA in AGO2 and Dicer knockdown RPE-1 cells. (**d**) Immunostaining of CENPC1 on RPE-1 metaphase chromosomes from Dicer or AGO2 knockdown cells. Split channels and merged view of the boxed area were shown below. ‘Dislocation’ refers both unequal loading of CENPC1 to centromeres and mislocation of CENPC1 to the chromosome arm. Quantification of CENPC1 dislocation was shown with bar figure (*n*>300 chromosomes per experiment). Scale bar represents 10 μm for **c** and 2 μm for **d**. **P-value<0.01. *P*-values were determined with two-tailed Student’s *t*-test. All data were from three repeats. Error bars represent s.d.

Considering that both AGO2 or Dicer knocking down and transfection of α-satellite RNA led to a similar phenotype of CENPC1 mislocation and chromosome lagging, we suspected that the RNAi pathway might function to keep the levels of α-satellite RNA in check. AGO2 RNA immunoprecipitation (RIP) assays with isolated metaphase chromosomes revealed that AGO2 interacted with α-satellite RNA on chromosomes (Figure 3a and Supplementary Figure S4C). Furthermore, knocking down AGO2 or Dicer resulted in increased levels of α-satellite RNA in both total RNA and chromosomal RNA samples (Figure 3b). FISH for α-satellite RNA revealed increased intensity, larger loci and mislocalization on mitotic metaphase chromosomes followed by applications of AGO2 or Dicer siRNA (Figure 3c). CENPC1 immunostaining of metaphase chromosomes revealed that CENPC1 localization was abnormal and dispersed with dislocation when AGO2 or Dicer was knocked down (Figure 3d). In addition, we noticed that the CENPC1 dislocation observed in Figure 3d was similar to that in Figure 2g with the transfection of exogenous α-satellite RNA, and both phenotypes were associated with an increased level of α-satellite RNA.

Collectively, our data demonstrated that AGO2 and Dicer regulated the amount and distribution of α-satellite RNA and further CENPC1 distribution on mitotic chromosomes. The total amount of CENPC1 was not altered (Figure 1c), but rather the protein mislocated on chromosomes in AGO2 or Dicer-deficient cells.

### The existence of ASAT siRNA and its role in chromosome segregation

**Figure 4:**
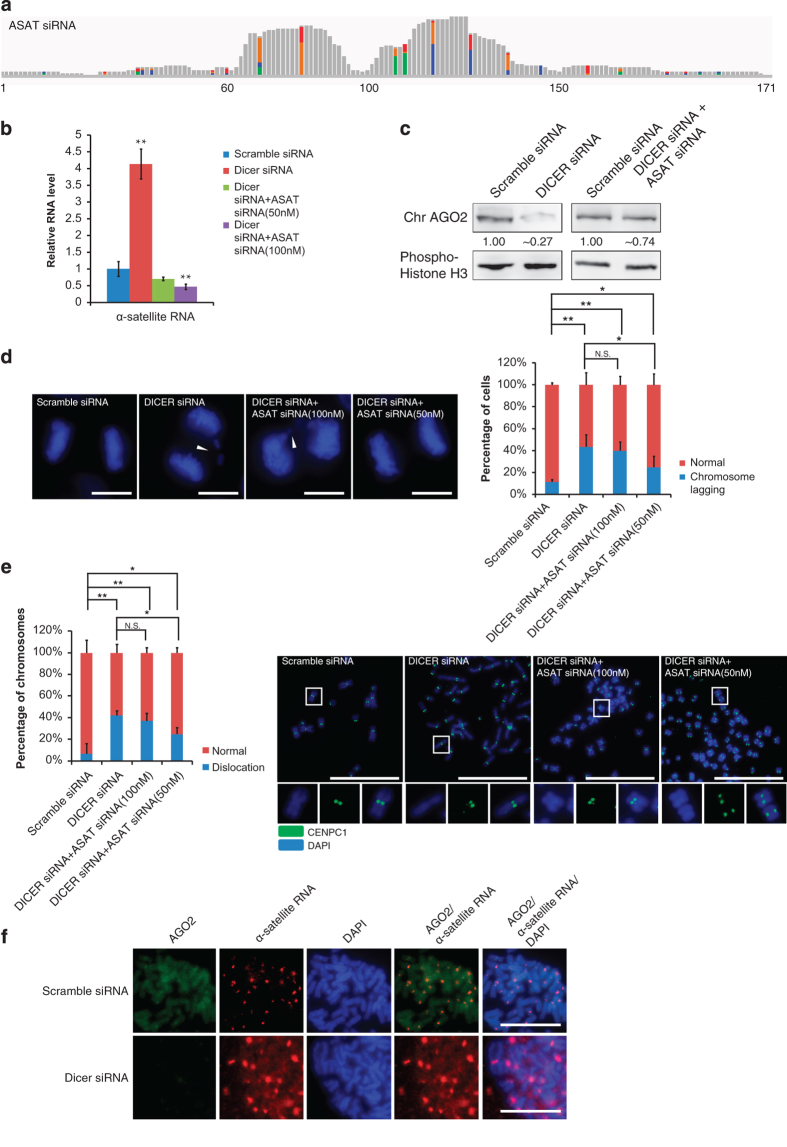
The existence of ASAT siRNA and its roles in chromosome segregation. (**a**) Bioinformatics analysis of alignment of ASAT siRNAs in HEK293 to the α-satellite RNA sequences. The height of the bars indicates the number of nucleotides covered by ASAT siRNAs. Gray line segment indicates a match, and other colors indicate mismatches. (**b**) Quantitative PCR to assess levels of α-satellite RNA in RPE-1. Scramble siRNA (negative control), Dicer siRNA (for Dicer knockdown) and Dicer siRNA+ASAT siRNA (Dicer knockdown with the application of exogenous ASAT siRNA). (**c**) Western blot assay with RPE-1 chromosome samples (treated with 100-nM ASAT siRNA and Dicer siRNA, with scramble siRNA as a control) to detect chromosomal AGO2 (Chr AGO2). Phospho-histone H3, a metaphase chromosomal protein, was used as a loading control. (**d**) Representative images of chromosome lagging (arrowheads) in RPE-1 cells during anaphase were shown for cells with various treatments. The statistics of the results were shown on the right (*n*>60 cells per experiment). (**e**) Immunostaining of CENPC1 on metaphase chromosomes from RPE-1 cells with various treatments. Enlarged images of the boxed area with split and merged channels were shown as inset for detailed view of CENPC1 localization. ‘Dislocation’ refers both unequal loading of CENP-C to centromeres and mislocation to the chromosome arm. Quantification of CENPC1 dislocation was shown with bar figure (*n*>300 chromosomes per experiment). (**f**) Co-staining of α-satellite RNA and AGO2 upon Dicer knockdown in RPE-1 cells. Scale bar represents 10 μm for **d**, and 5 μm for **e** and **f**. *P-value <0.05; **P-value <0.01. *P*-values were determined with two-tailed Student’s *t*-test. All data were from three repeats. Error bars represent s.d. DAPI, 4′, 6-diaminodino-2-phenylinodole. N.S., not significant.

How does the RNAi pathway manage level and distribution of chromosomal α-satellite RNA? Considering that AGO2 needs the guidance from small RNAs, we sequenced HEK293 cells for small RNAs and analyzed the sequencing data along with small RNA reads from other human cells such as MCF7 and ESC [35, 36]. We found that endo-siRNAs mapped to the 171-base pair (bp) α-satellite repeat element, and most reads mapped roughly 60–80 and 90–120 bp regions of the α-satellite repeat element (Figure 4a and Supplementary Figure S5A). We termed these siRNAs α-satellite siRNA (ASAT siRNA).

Co-transfection of 50 or 100 nM *in *vitro synthesized ASAT siRNAs could reverse the abnormal increase in α-satellite RNA seen under Dicer knockdown (Figure 4b and Supplementary Figure S5B). Actually, 100-nM exogenous ASAT siRNA under Dicer knockdown resulted in a significantly decreased level of α-satellite RNA compared with the normal level (scramble siRNA control), indicating that the concentration of 100 nM might be too high. Indeed, 50-nM ASAT siRNA under Dicer knockdown resulted in a slightly decreased (but not statistically significant) level of α-satellite RNA (Figure 4b).

As our data showed that AGO2 is a chromosomal protein (Figure 1d and e), we examined the chromosomal AGO2 (chr AGO2) level in the absence of Dicer (Figure 4c). Dicer knockdown decreased chromosomal AGO2 level significantly, and exogenous ASAT siRNAs could restore the level of AGO2 protein on metaphase chromosomes upon Dicer knockdown (Figure 4c). Furthermore, the exogenous ASAT siRNAs could partially rescue the chromosome lagging phenotype and restore CENPC1 localization in Dicer knockdown cells (Figure 4d and e). We also analyzed the chromosomal localization of AGO2 and found that AGO2 IF signals were decreased, whereas FISH signals of α-satellite RNAs were increased at both centromere and chromosome arms upon Dicer knockdown (Figure 4f). A colocalization at the centromere for AGO2 and the overexpressed α-satellite RNAs upon dicer knockdown can still be observed, and AGO2 was presumably guided by limited ASAT siRNA that is still available upon Dicer knockdown.

Taken together, these results support our hypothesis that AGO2 guided by ASAT siRNAs generated by Dicer controls the level and distribution of α-satellite RNAs and further CENPC1 localization at the centromere.

### Slicer activity of AGO2 is essential for its role in chromosome segregation

**Figure 5:**
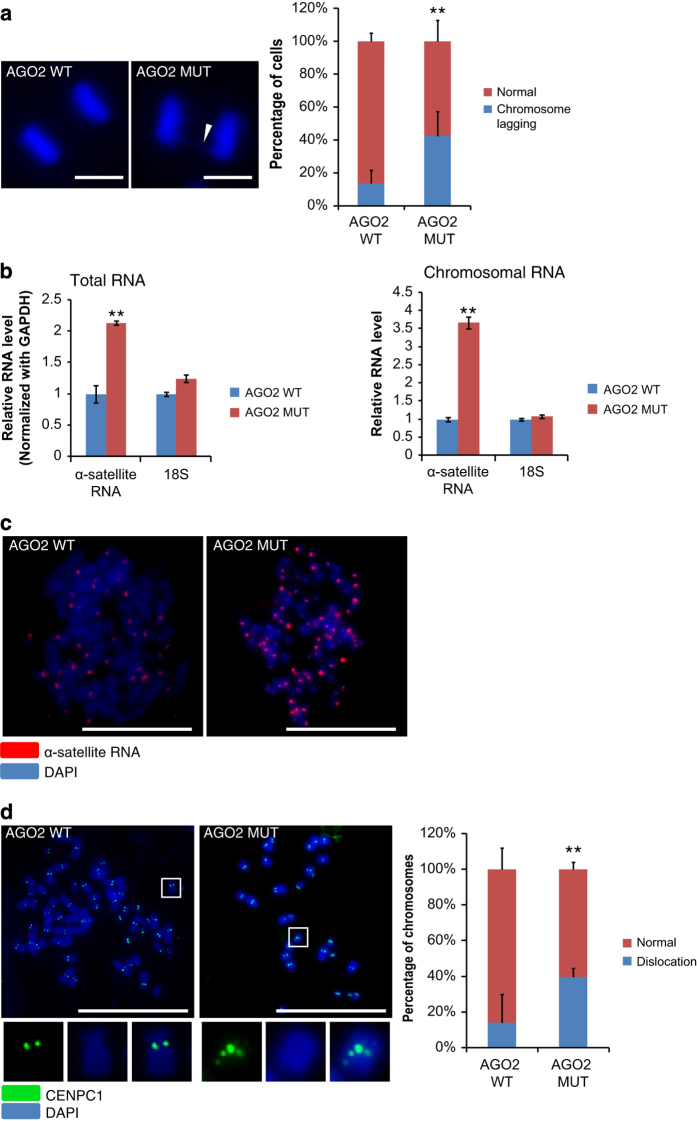
Slicer activity of AGO2 is essential for its role in chromosome segregation. (**a**) Representative images of chromosome lagging (arrowheads) during anaphase were shown, and the statistics of the results were shown on the right (*n*>60 cells per experiment). AGO2 WT (cells transfected with wild-type AGO2); AGO2 MUT (cells transfected with mutant AGO2 without the enzymatic slicing activity). (**b**) Quantitative PCR to assess the α-satellite RNA level using total or chromosomal RNA of RPE-1 cells. 18 S rRNA was examined as a negative control. (**c**) RNA-FISH assay of α-satellite RNA with RPE-1 metaphase chromosomes. (**d**) Representative images of immunostaining of CENPC1 on RPE-1 metaphase chromosomes. Enlarged images of the boxed area were shown as inset for detailed view of CENPC1 localization. ‘Dislocation’ to refer both unequal loading of CENPC1 to centromeres and mislocation of CENPC1 to the chromosome arm. Quantification of CENPC1 dislocation was shown with bar figure (*n*>300 chromosomes per experiment). Scale bar represents 10 *μ*m in **a**, **c** and **d**. RPE-1 cells were used for all panels. **P-value<0.01. *P*-values were determined with two-tailed Student’s *t*-test. All data were from three repeats. Error bars represent s.d.

Considering the involvement of ASAT siRNAs in the control of α-satellite RNA and the requirement of AGO2 rather than the other Argonautes in mitotic chromosome segregation, we speculated that the catalytic function of AGO2 might be crucial. Actually, AGO2 is the only member possessing slicer activity to cleave the substrate RNA under the guidance of siRNA among the four mammalian Argonautes [37]. To investigate this issue, we transfected a mutant AGO2, which was devoid of the slicer activity in RPE-1 cells, with a wild-type AGO2 as the control (Supplementary Figure S6) [37]. This mutant AGO2 can function like a dominant-negative protein [30]. We observed higher incidence of chromosome lagging in cells with AGO2 mutant (Figure 5a). Levels of α-satellite RNA were significantly increased in both total and chromosomal RNA samples upon overexpression of the AGO2 mutant (Figure 5b). In consistence with this increase, α-satellite RNA signal on mitotic chromosomes became brighter and dispersed in cells with AGO2 mutant (Figure 5c). Furthermore, CENPC1 immunostaining was aberrant with mislocation on chromosomes in samples with AGO2 mutant (Figure 5d).

Taken together, we concluded that the slicer activity of AGO2 was essential for the RNAi machinery to participate in mammalian chromosome segregation, and thus a siRNA pathway rather than a miRNA pathway was involved.

## Discussion

**Figure 6:**
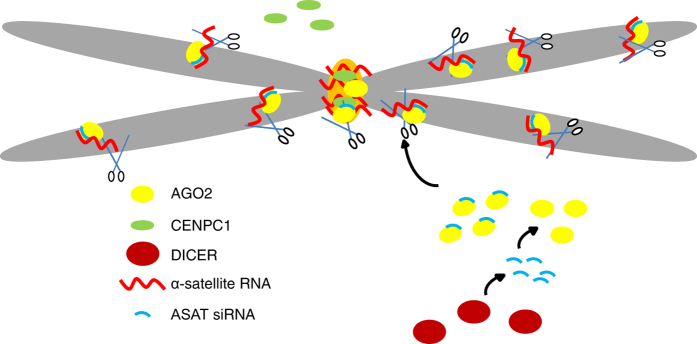
The model. Dicer generates endo-ASAT siRNAs, and then these ASAT siRNAs guide AGO2 to target the α-satellite RNAs on mitotic chromosomes. With the slicer activity of AGO2, α-satellite RNAs on chromosome arms are eliminated, and overaccumulation of α-satellite RNAs on the centromere is prevented. The right amount and localization of α-satellite RNAs on the core centromere ensure proper deposition of centromere/kinetochore proteins such as CENPC1. Thus, the RNAi machinery is essential for the formation of functional centromere/kinetochore and the final chromosome segregation in mammalian cells.

Our data support a model in which the RNAi machinery participates in chromosome segregation by directly keeping the level and distribution of α-satellite RNA on mitotic chromosomes in check (Figure 6). Dicer generates endo-ASAT siRNAs presumably from double-stranded RNAs derived from the centromeric repeats, as transcripts from both directions of the repeats are known to exist in mammalian cells [34]. These endo-ASAT siRNAs then guide AGO2 to target the α-satellite RNAs on mitotic chromosomes. With the slicer activity of AGO2, α-satellite RNAs that mislocalize on chromosome arms and overaccumulate on the centromere are eliminated. The right amount and localization of α-satellite RNAs on the centromere ensure proper deposition of CENPC1 and maybe other components required for the proper formation and function of centromere/kinetochore on mitotic chromosomes. The RNAi pathway is required to keep the fine balancing of α-satellite RNAs, as our data actually show that either too much, too little or dislocation of these RNAs on the chromosome would lead to chromosome lagging. Most previous analyses filtered out small RNAs originated from genomic repeats, and we might be the first to realize the presence of ASAT siRNAs in human cells. The identification of ASAT siRNAs as the guide for AGO2 to target α-satellite RNAs directly links the RNAi pathway to mammalian chromosome segregation.

CENP-C is an essential protein for the assembly of kinetochore. In a recent publication with chicken cells in which the endogenous centromere of a chromosome was removed, the full length or the C terminus of CENP-C protein tethered at a non-centromere site could induce the formation of an ectopic kinetochore with the full complement of kinetochore proteins [38]. Ectopic tethering of the CENP-C N terminus also generated functional kinetochores, although these kinetochores lacked some constitutive centromere-associated proteins [38].

In human cells, the mislocalized α-satellite RNAs on chromosomes upon Dicer or AGO2 knockdown could recruit CENP-C via its RNA-binding motif. Some other centromere/kinetochore proteins might also be recruited by interacting with CENP-C, and eventually these malformed structures would interfere with the precise process of chromosome segregation and lead to chromosome lagging. α-satellite RNAs at the centromere/kinetochore have to be maintained at the right level presumably by the balancing between local transcription at the centromere and the RNAi machinery. However, we have to point out that our data about CENPC1 dislocation upon Dicer knockdown are different from a report with a chicken–human hybrid DT40 cell line, in which loss of Dicer (Dicer null) resulted in accumulation of abnormal mitotic cells, but the localization of CENP-C was normal [20]. The reason might be that DT40 is a chicken–human hybrid cell, which is different from human cell, or the amount at the centromere rather than the localization of CENP-C was misregulated in the hybrid cell. The level of Dicer protein is also different between our research and the DT40 study, as we used siRNA to knockdown Dicer.

Another point of particular interest is the localization of AGO2 on mitotic chromosomes (Figure 1d and e and Supplementary Figure S3E). Locations and activity of Dicer, AGO2 and the RISC complex are subjected to dynamic regulation in eukaryotic cells [22, 23, 39, 40]. The localization of AGO2 on metaphase chromosome is important in managing α-satellite RNAs, but may also be with vital physiological functions in keeping other RNA transcripts in check, as our data show that a myriad of ncRNAs derived from repeats are present on metaphase chromosomes (Figure 2a). It seems that AGO2 but not the other three Argonautes is involved in mammalian chromosome segregation. Interestingly, among the multiple *Caenorhabditis elegans* Argonaute proteins, only CSR-1 is known to be required for chromosome segregation [41].

In *Drosophila*, both Dicer-2 and Ago-2 are required for chromosomal localization of the RNA helicase Belle, and Dicer-2 and Ago-2 are related to chromosome segregation by promoting proper localization of proteins such as Belle [24]. This regulatory mechanism may be conserved in human cells. Human Dicer and the Belle homolog DDX3 interact with each other, and Dicer knockdown results in dislocation of DDX3 and defective chromosome segregation in HeLa cells [24]. We suspect that Dicer and AGO2 in human cells are required for the correct amount and distribution of both ncRNAs (for example, α-satellite RNAs) and proteins (for example, CENPC1 and DDX3). Recent study has shown that satellite III (SAT III) RNAs serve as an important component in the kinetochore assembly, in which SAT III RNAs bind to kinetochore component CENP-C, and is necessary for correct localization of centromeric proteins such as CENP-C in *Drosophila* [26, 42]. It is possible that the *Drosophila* RNAi machinery may have a role in chromosome segregation by directly managing SAT III RNAs, just similar to what we report here for the mammalian RNAi pathway. It is also known that centromere-encoded RNAs are components of the kinetochore in maize [43]. Whether and how the RNAi machinery manages centromeric ncRNAs involved in chromosome segregation in other sectors of eukaryotic organisms remain to be investigated.

Murine cells with abnormal accumulation of minor satellite RNAs showed chromosome lagging [16], in a way similar to phenotype seen for human cells with higher levels of α-satellite RNAs (Figure 2e). Despite the distinction in the actual centromere repeat sequences, mammalian cells may share similar molecular mechanisms in regulating centromere/kinetochore formation with ncRNAs derived from centromeric repeats. A recent publication demonstrates that minor satellite RNA can even have roles *in trans* to regulate telomerase via interacting with Aurora Kinase B in mouse embryonic stem cells [10]. It would be interesting to investigate whether the mammalian RNAi machinery also regulates this kind of *trans* effect of ncRNAs transcribed from centromeric repeats.

The canonical RNAi pathway mediated by miRNAs or siRNAs is viewed mainly as a post-transcriptional regulatory mechanism of gene expression in mammalian cells [22, 23, 39]. Here we have integrated lines of evidence about the involvement of ncRNAs and endo-siRNA pathway in the function of centromere/kinetochore. With our data, it is clear that the mammalian RNAi machinery can not only have roles in gene expression but also participates in cellular behaviors such as mitotic chromosome segregation by directly managing vital ncRNAs involved in.

## Materials and Methods

### Isolation of metaphase chromosomes

Cells were incubated with colchicine (20 ng ml^−1^) for 15 h, resulting in a mitotic index of 60–70%. Mitotic chromosomes were isolated following the protocol from Hayashihara *et al. *[31] (Supplementary Figure S2A and C) . Metaphase chromosomes isolated with this method were used for western blots and isolation of chromosomal RNAs (for real-time PCR, northern blots and RNA sequencing).

### Preparation of nuclei, cytoplasm and whole-cell lysis

Nuclei and cytoplasm were prepared with the KeyGen Nuclei Isolation Kit (Cat number: KGA826; Nanjing, China) following the protocol provided. For whole-cell lysis preparation, we collected cells and added with sample buffer (125 mM Tris pH 6.8, 25% (v/v) glycerol, 4% sodium dodecyl sulfate (SDS), 10% β-mercaptoethanol). Western blots were performed with samples from the same number of cells containing the corresponding fraction. Only one-sixth of the WCL (whole-cell lyses) and CYTO (cytoplasmic) was loaded to avoid saturation of the corresponding bands (Figure 1d).

### Preparation of metaphase chromosome spreading by dropping

Cells were synchronized with colchicine (100 ng ml^−1^) for 4 h and collected after phosphate-buffered saline washing. Collected cells were treated with hypotonic solution (75 mM KCl) for 30 min at 37 °C. Then, hypotonic cells were collected and fixed with methanol: glacial acetic acid (3:1) solution twice for 1 h. Fixed cells were spun down and resuspended with methanol: glacial acetic acid solution. A small quantity of cell suspension was held vertically ~1 m above the slide (cold). Then a single drop was released from this height on the slide. Slides were washed with phosphate-buffered saline twice and then used for immunostaining and FISH.

### RIP assay

RIP was performed following the protocol from Li *et al.* 2015 [44]. Cells were washed and sonicated in lysis buffer (50 mM Tris-HCl at pH 8, 150 mM NaCl, 2 mM EDTA at pH 8, 1% NP-40, 0.5% sodium deoxycholate and 0.1% SDS, protease inhibitors) containing RNase inhibitors (Thermo Scientific, Waltham, MA, USA) and bovine serum albumin carried out as previously described with some modifications. RIP was performed with the relevant antibodies (anti-AGO2, 1:1 000 dilution and IgG as control) and captured with protein G Sepharose 4 Fast Flow suspension (GE Amersham, Little Chalfont, UK). The beads were performed with proteinase K (Sangon, Shanghai, China) digestion for 1 h followed by RNA purification by TRIzol reagent (Life Technologies, Waltham, MA, USA). Real-time quantitative (qPCR) or semi-quantitative reverse-transcription PCR (RT-PCR) was used to examine the RNA yielded, and primers used for these PCR reactions are listed in Supplementary Table S1.

### RNA sequencing and bioinformatic analysis

For HeLa chromosomal RNA high-throughput sequencing, the libraries were prepared following the manufacturer's instructions and applied to Illumina GAIIx system for 80-nt single-end sequencing by ABlife (Wuhan, China). For chromosomal RNA repeats dividing, we filtered the reads to the reference genome downloaded from UCSC (HG18). Small RNA high-throughput sequencing was performed with Illumina GAII 2000 (Illumina, San Diego, CA, USA) with total RNA isolated from HEK293 cells. Small RNA reads for MCF7 cell (GEO Accession: GSM402329) and ES cell (GEO Accession: GSM450239) were also analyzed for ASAT siRNAs [35, 36]. In brief, small RNA sequencing reads were mapped to the α-satellite region in tolerance of two mismatches. Upon acceptance, the data will be deposited into the NCBI Gene Expression Omnibus database, and the accession code will then be provided.

### Northern blot analysis

Digoxin-labeled RNA probes were transcribed with the corresponding insertion in the T vector as a template following the manual of DIG Northern Starter Kit (Roche, Basel, Switzerland). Total RNA and chromosomal RNA (both were 7.5 μg) were loaded on a precast gel (Bio-Rad, Hercules, CA, USA) containing 15% urea and were ran for 3 h in 1× TBE buffer. RNA was transferred onto Hybond-N+ membrane (GE Healthcare, Little Chalfont, UK) by electrophoretic transfer. Hybridization was performed with probes (final concentration 100 ng ml^−1^) at 62 °C overnight. Membranes were stringently washed twice in wash buffer (2× SSC, 0.1% SDS at 37 °C and 0.1× SSC, 0.1% SDS) at 62 °C for 30 min, respectively, and detection was performed following the protocol provided (Roche, DIG Northern Starter Kit). Images were taken with LAS-4000mini Image Reader (GE Healthcare). Information for oligos used is provided in Supplementary Table S1.

### Flow cytometry

The separation profiles of cleared mitotic chromosome fraction and nucleus fraction were confirmed using flow cytometry after staining with propidium iodide (35 μg ml^−1^). Flow cytometry was performed with FACSCalibur (BD Biosciences, Franklin Lakes, NJ, USA) and FlowJo 7.6 software (FlowJO LLC, Ashland, OR, USA).

### Plasmids and plasmid construction

All plasmids were constructed with restriction enzyme digestion and ligation, and were sequenced for confirmation. For AGO2 mutant plasmid construction, 633 and 634 sites of AGO2 were mutated from Q and H to R and A, respectively. The shRNA plasmids for knockdown of AGO1 mRNA (TRCN0000009625), AGO3 mRNA (TRCN0000009636) and AGO4 mRNA (TRCN0000009640) with negative shRNA control (SHC002) were obtained from the MISSION shRNA Library (Sigma, St Louis, MO, USA). Information for oligos used is provided in Supplementary Table S1.

### FISH

RNA probes were prepared by SP6 (Thermo Scientific) with the corresponding insertion in the T vector as a template, and were labeled with Alexa Fluor546 by using the ULYSIS Nucleic Acid Labeling Kit (Invitrogen, Waltham, MA, USA) following the manual provided. The fixed cells and RNA probes (final concentration 2 ng μl^−1^) were denatured at 80 °C for 10 min, and then incubated at 37 °C for 15–17 h with yeast tRNA blocking. Slides were washed twice with 2× SSC at 45 °C for 10 min. Images were taken with Zeiss Imager A2 microscope (Zeiss, Oberkochen, Germany). Information for oligos used is provided in Supplementary Table S1.

### IF staining

For immunostaining, slides with cleared mitotic chromosomes or synchronized cells were incubated with antibody against CENPC1 (Abcam, Cambridge, UK; 1:100 dilution, cat no. ab50974), CENP-B (Abcam, 1:100 dilution, cat no. ab25734), Phospho-Histone H3 (Cell Signaling Technology, 1:100 dilution, cat no. 9706), AGO2 (Sigma, 1:100 dilution, cat no. SAB4200085), Dicer (Sigma, 1:100 dilution, cat no. SAB4200087) or ACTB (Abcam, 1:100 dilution, cat no. ab8227) followed with incubation with Alexa Fluor488 or 546 labeled secondary antibody (Abcam, 1:200 dilution cat nos ab150105 and ab150074).

### Cell culture and transfection of plasmids, siRNA and α-satellite RNA

HEK293, HeLa and RPE-1 were cultured under standard conditions with DMEM plus 10% fetal bovine serum at 37 °C and 5% CO_2_. MEF (DGCR8 null) cells were cultured under standard conditions with DMEM plus 10% fetal bovine serum and 1% NEAA at 37 °C and 5% CO_2_. RPE-1 cells were used in essentially all the FISH and IF experiments because they are diploid. Plasmids and siRNA transfection was performed with Lipofectamine 2000 (Invitrogen) according to the supplier’s instructions (CENPC1 siRNA-1, 2, 100 nM; AGO2 siRNA-1, 2, 100 nM; Dicer siRNA-1, 2, 100 nM; ASAT siRNA-1, 2, 50 or 100 nM). α-satellite RNA and Line 1 repeat-derived RNA (final concentration 5 ng μl^−1^) transfections were performed with electroporation using Nucleofection (Lonza, Basel, switzerland) according to the attached instructions. Information for oligos used is provided in Supplementary Table S1.

### Antibodies and western blot analysis

For western blots, samples were separated on SDS-polyacrylamide gel electrophoresis gels and then transferred to Polyvinylidene fluoride membranes (Millipore, Billerica, MA, USA). Membranes were processed following the ECL western blotting protocol (GE Healthcare). These antibodies were used in western blots: anti-CENPC1 (Abcam, 1:1 000 dilution, cat no. ab50974), anti-Phospho-Histone H3 (Cell Signaling Technology, 1:1 000 dilution, cat no. 9706), anti-ACTB (Abcam, 1:1 000 dilution, cat no. ab8227), anti-Dicer (Sigma, 1:2 000 dilution, cat no. SAB4200087), anti-GAPDH (Cell Signaling Technology, Danvers, MA, USA, 1:2 000 dilution, cat no. 3683), anti-AGO2 (Sigma, 1:2 000 dilution, cat no. SAB4200085) and anti-HDAC2 (Cell Signaling Technology, 1:1 000 dilution, cat no. 5113). Images were taken with LAS-4000mini Image Reader (GE Healthcare).

### PCR reactions

Total and chromosomal RNAs were purified from cells and chromosomes by using the TRIzol reagent (Invitrogen) with the attached instructions. Complementary DNA for qRT-PCR was synthesized from total and chromosomal RNA using the GoScript Reverse Transcription System (Promega, Madison, WI, USA) according to the attached procedures using random hexamer primer or oligo dT. qPCR was performed with Platinum SYBR Green qPCR Supermix UDG (Invitrogen) on PikoReal Real-Time PCR System (Thermo Scientific) according to the standard procedures. For semi-quantitative PCR and semi-quantitative RT-PCR, PCR cycle numbers were set between 20 and 25 to avoid saturation of PCR reactions. Information for oligos used is provided in Supplementary Table S1.

### Statistical analysis

Relative mRNA level was calculated by ∆∆*C*_q_ method. The values reported in the graphs represent the average of three independent experiments, with error bars showing s.d. After analysis of variance by F-test, the statistical significance and *P*-value were evaluated by Student’s *t*-test.
