# Supplementary material for: RNAi pathway participates in chromosome segregation in mammalian cells
Source: Cell Discov. 2015 Oct 20;1:15029–. doi: 10.1038/celldisc.2015.29 (PMC4860838; doi:10.1038/celldisc.2015.29)
Supplement: Supplementary Figure Legends [file celldisc201529-s1.doc]

**RNAi pathway participates into chromosome segregation in mammalian cells**

Chuan Huang, Xiaolin Wang, Xu Liu, Shuhuan Cao, Ge Shan

**SUPPLEMENTARY INFORMATION**

Supplementary Figure S1-6

**Figure Legends of Supplementary Figures**

**Figure S1 | Effect and efficiency of AGO2 and Dicer knockdown.** (**A**) The knockdown efficiency of AGO2 and Dicer in RPE-1 cells examined with quantitative PCR and western blot assay. Two sets of siRNAs were checked. (**B**) Knocking down of AGO2, Dicer and CENPC1 with siRNAs in Hela cells. Representative images of chromosome lagging (arrowheads) during anaphase were shown. Statistics of the results were shown with bar figure to the right (n > 60 cells per experiment). (**C**) The knockdown efficiency of AGO2 and Dicer with the corresponding siRNA in Hela cells. (**D**) The knockdown of AGO2 or Dicer did not show mutual effect on the mRNA level of each other. (**E**) Levels of mature forms of miR-221 and miR-452 upon Dicer or AGO2 knockdown in HeLa cells. (**F**) Two randomly selected miRNAs, miR-221 and miR-452, could not be detected in DGCR8-null cells. (**G**) The knockdown efficiency of AGO2, Dicer and CENPC1 with the corresponding siRNA in MEF (DGCR8-null) cells measured by quantitative PCR. Scale bar represents 10 µm. ** *P* value < 0.01. *P* values were determined with two-tailed Student’s *t*-test. All data were from three repeats. Error bars represent S. D.

**Figure S2 | The other three Argonautes are not involved in chromosome segregation.** (**A**) Representative images of AGO1, AGO3 and AGO4 knockdown cells during anaphase. The statistics of chromosome lagging were shown to the right (n > 60 cells per experiment). (**B**) The knockdown efficiency of AGO1, AGO3 and AGO4 with the corresponding shRNA measured by quantitative PCR. Scale bar represents 10 µm. * P value < 0.05; ** P value < 0.01. P values were determined with two-tailed Student’s t-test. All data were from three repeats. Error bars represent S. D.

**Figure S3 | Metaphase chromosome isolation and Argonautes on chromosome.** (**A**) Brief experimental strategy for batch isolation of metaphase chromosomes with gradient centrifugation. (**B**) Flow cytometry assay showed the relative enrichment of chromosomes in the chromosome fraction. (**C**) Immunostaining of phos-histone H3 (marker of mitotic chromosome) and ACTB (Beta-Actin, negative control) on the isolated mitotic metaphase chromosomes. (**D**) The overexpressed GFP tagged AGO1, AGO3 and AGO4 were not detected on mitotic chromosomes (CHR). GFP channel essentially gave no signal in all CHR samples. The expression of GFP tagged AGO1, AGO3 and AGO4 in transfected cells (CELL) was shown in the bottom. (**E**) Representative images for AGO2 immunostaining for cells at different mitotic phases. The arrows point to the nucleus or chromosome to show relatively lack of AGO2 in the interphase nucleus and presence of AGO2 on the chromosomes and telophase nucleus. Scale bar represents 10 µm. Hela cells were used in all panels.

**Figure S4 | Transfection of α-satellite RNA and its interaction with AGO2.** (**A**) The brief diagram of experimental setup for results shown in Figure **2f** and **2g**. (**B**) The successful transfection of α-satellite RNA into cell nuclei was verified with quantitative PCR. (**C**) AGO2 RIP assay with HEK293 cells followed with semi-quantitative PCR. ** *P* value < 0.01. *P* values were determined with two-tailed Student’s *t*-test. All data were from three repeats. Error bars represent S. D.

**Figure S5 | ASAT siRNAs in human cells and transfection of exogenous ASAT siRNAs.** (**A**) Bioinformatic analysis indicated the counts of different sequences of ASAT siRNAs mapped to the full length of α-satellite RNA in human MCF7 and ES cells (RNA-seq data from ref. 35, 36). (**B**) The brief schematic diagram for experimental setup for results shown in Fig. **4b-4e**.

**Figure S6 | AGO2 mutant.** (**A**) Schematic diagrams for c-myc tagged constructs of AGO2 wild-type and AGO2 mutant. (**B**) Western blot assay with antibody against AGO2 or c-myc tag for chromosome samples from RPE-1 cells transfected with AGO2 wild-type (WT) and AGO2 mutant (MUT) plasmid respectively. Phos-histone H3, a marker of mitotic chromosome, was used as a loading control.
