# Supplementary material for: RNAi pathway participates in chromosome segregation in mammalian cells
Source: Cell Discov. 2015 Oct 20;1:15029–. doi: 10.1038/celldisc.2015.29 (PMC4860838; doi:10.1038/celldisc.2015.29)

Supplementary Information
Figure S1

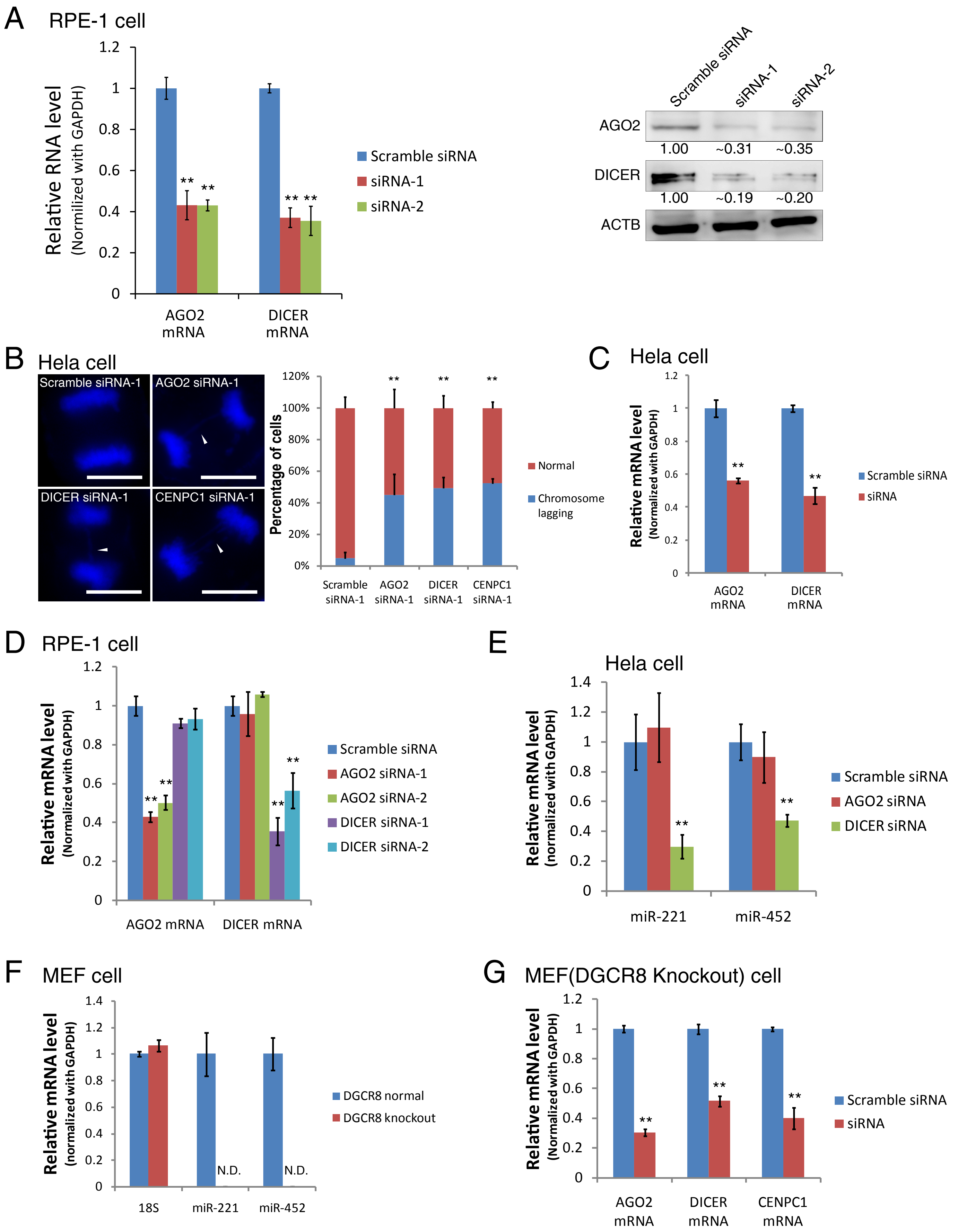

Supplement: Supplementary Figure S1 [file celldisc201529-s2.pdf]
