# Supplementary figures and images for: RNAi pathway participates in chromosome segregation in mammalian cells
Source: Cell Discov. 2015 Oct 20;1:15029–. doi: 10.1038/celldisc.2015.29 (PMC4860838; doi:10.1038/celldisc.2015.29)

Figure S2

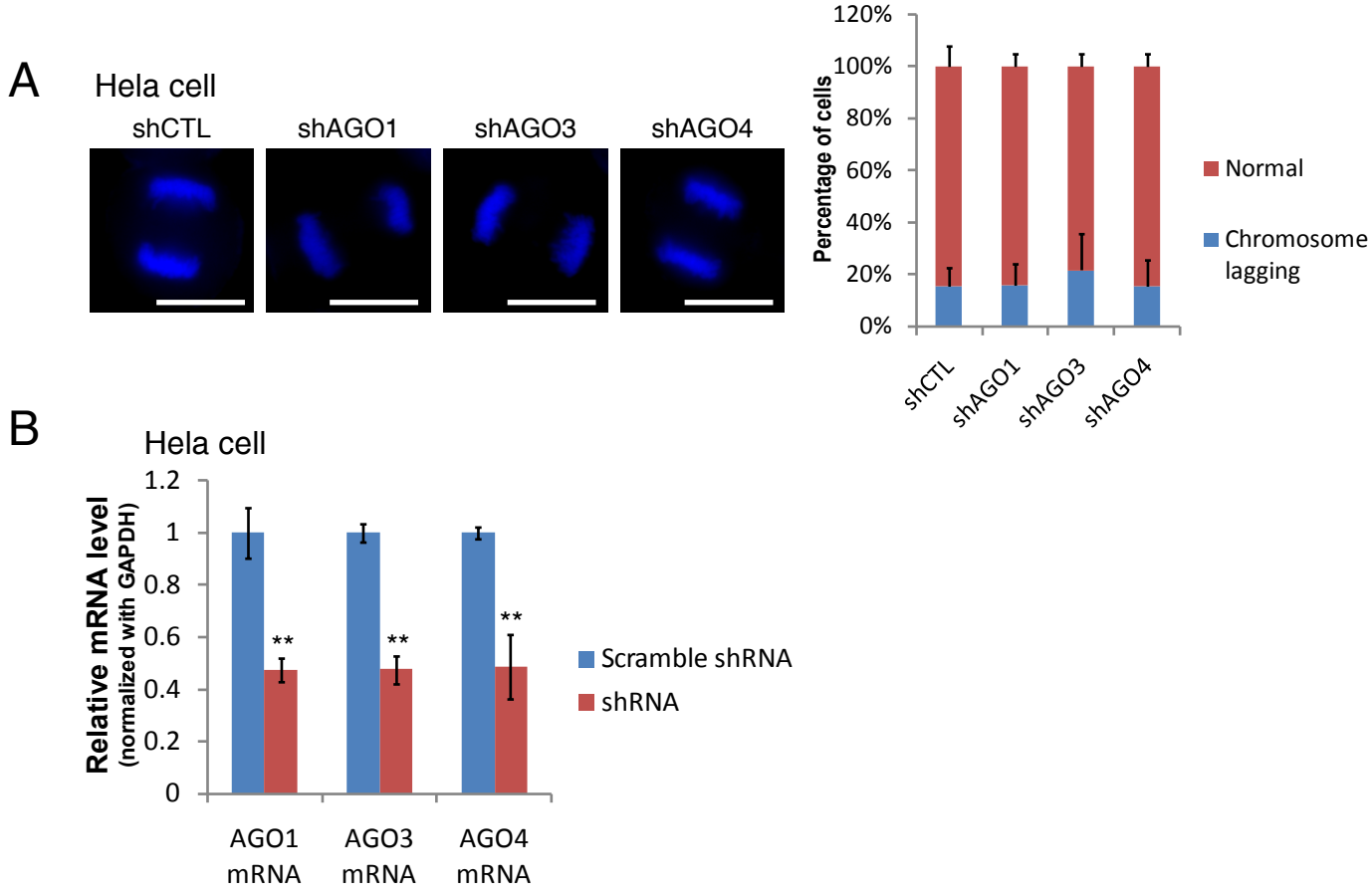

Supplement: Supplementary Figure S2 [file celldisc201529-s3.pdf]

# Figure S3

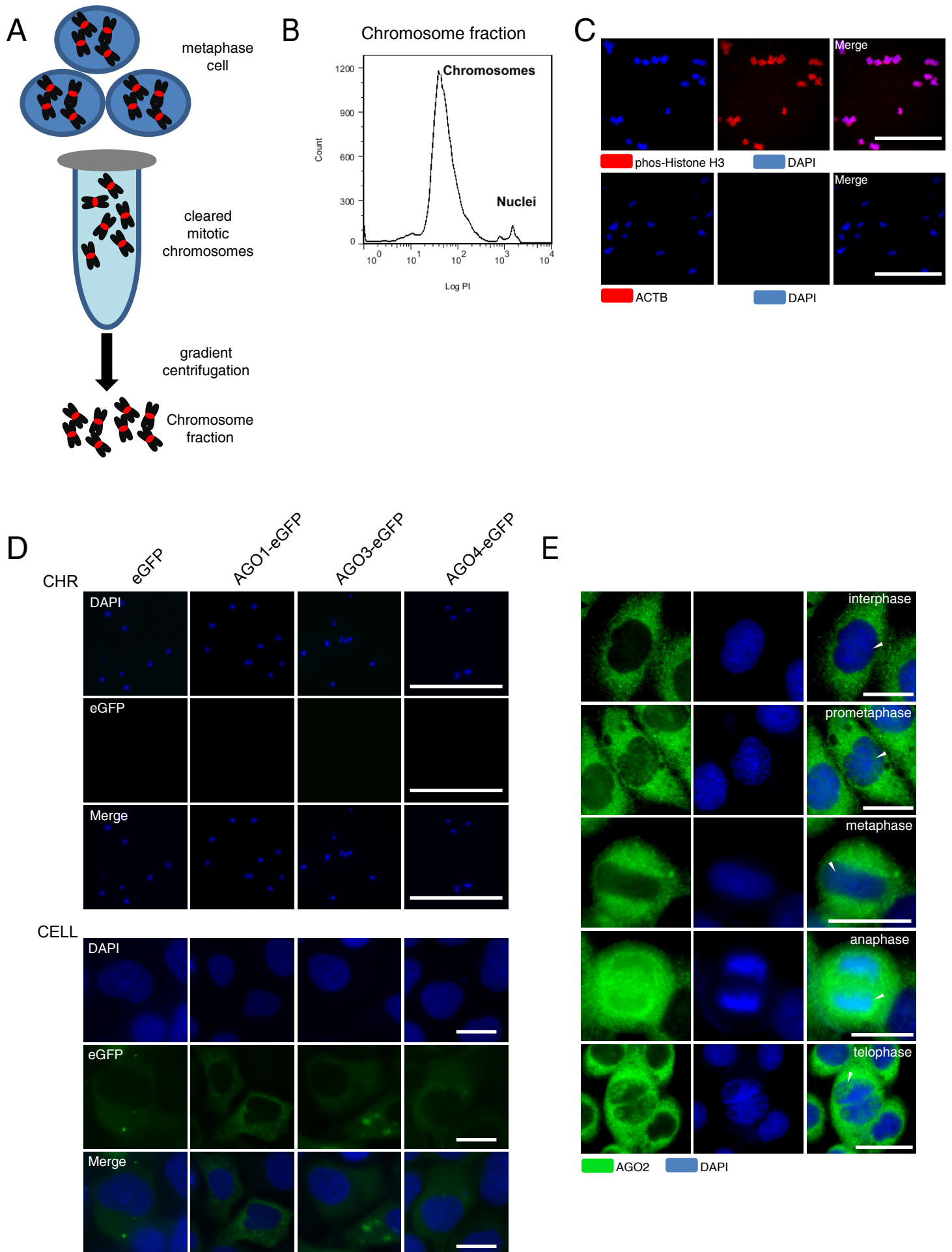

Supplement: Supplementary Figure S3 [file celldisc201529-s4.pdf]

Figure S4

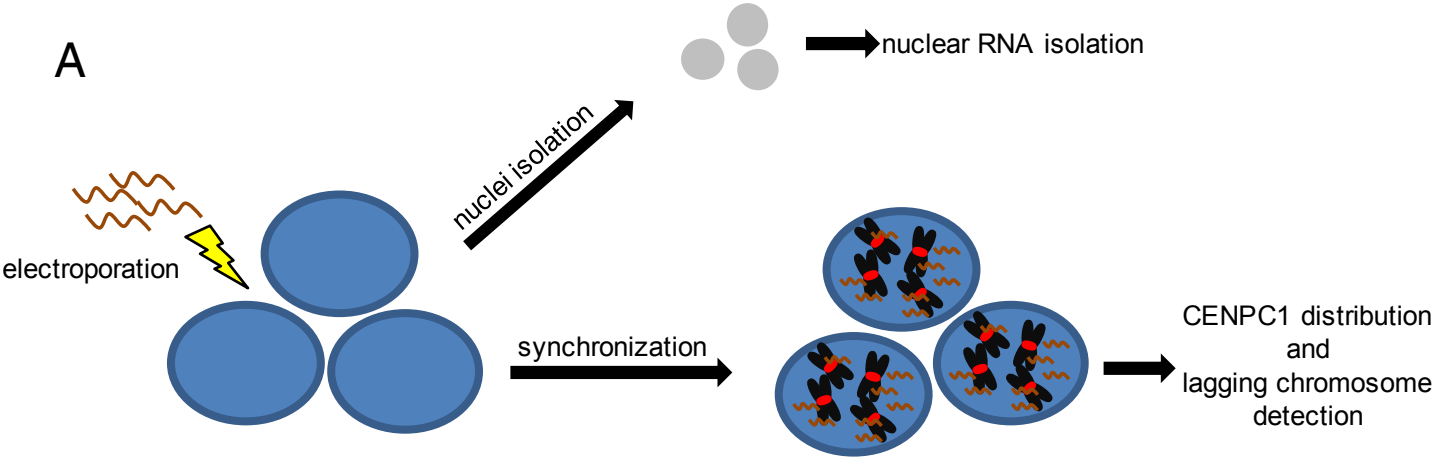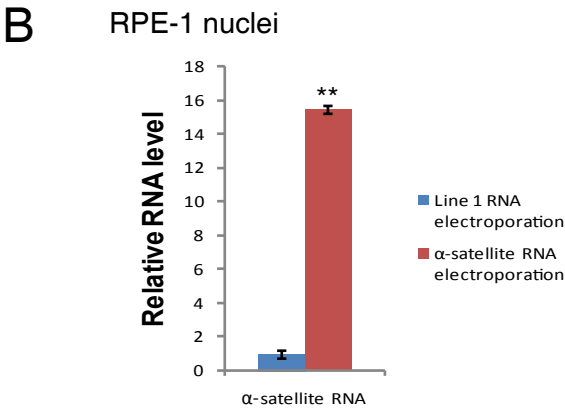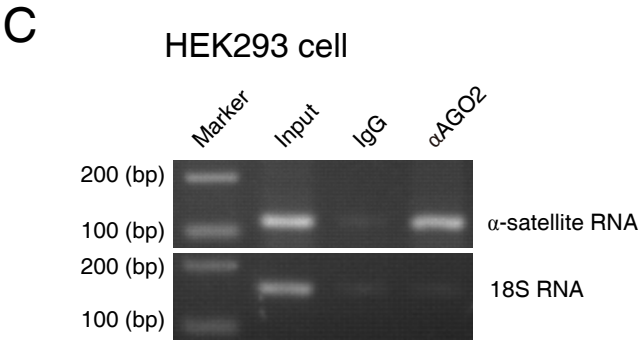

Supplement: Supplementary Figure S4 [file celldisc201529-s5.pdf]

# Figure S5

## A

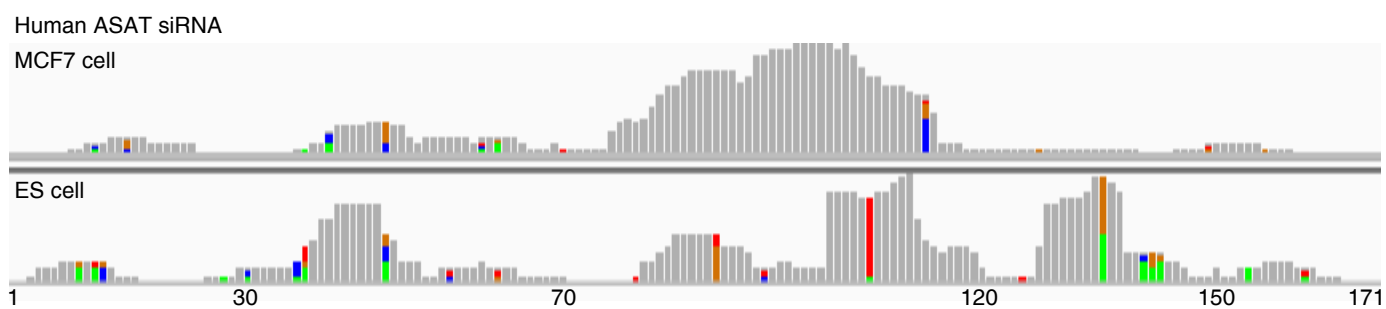

## B

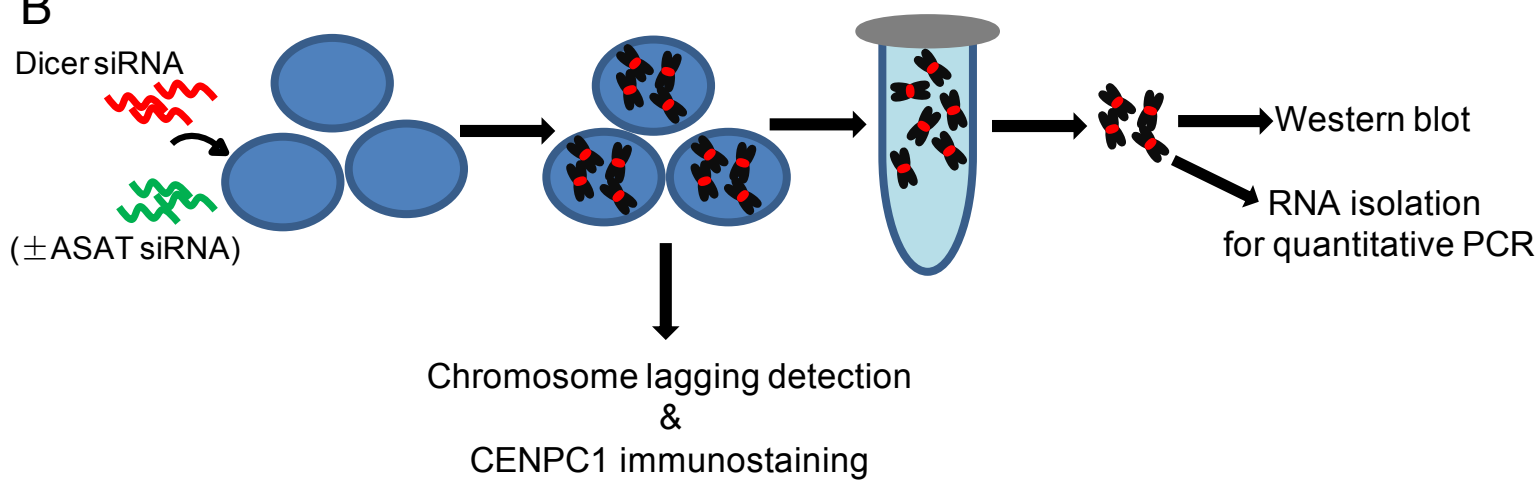

Supplement: Supplementary Figure S5 [file celldisc201529-s6.pdf]

Figure S6

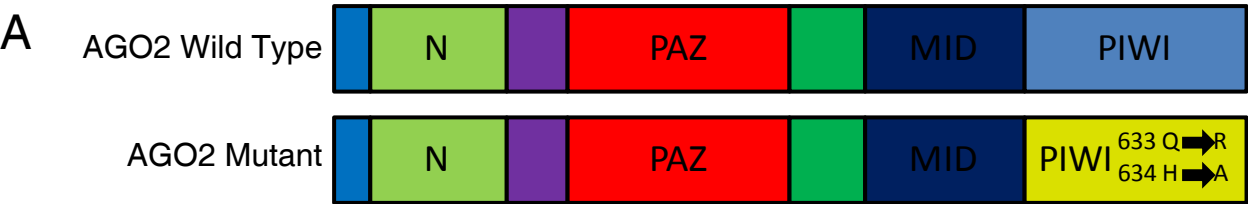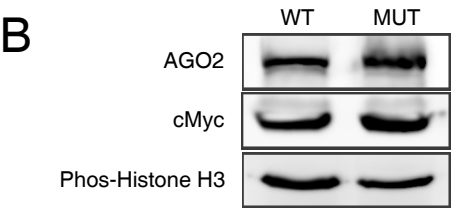

Supplement: Supplementary Figure S6 [file celldisc201529-s7.pdf]
