# Supplementary material for: RNAi pathway participates in chromosome segregation in mammalian cells
Source: Cell Discov. 2015 Oct 20;1:15029–. doi: 10.1038/celldisc.2015.29 (PMC4860838; doi:10.1038/celldisc.2015.29)
Supplement: Supplementary Table S1 [file celldisc201529-s8.pdf]

Supplementary Table 1

Oligos used in this study

Oligo name	Sequence (5' - 3')	Purpose	Amplicon
AGO1_F	GGCTCGAGTATATGGGATGGAAGCGGGA	plasmid construction for AGO1	2598
AGO1_R	GGGGATCCTCAAGCGAAGTACATGGTGC		
AGO3_F	GGCTCGAGCATGAATGGAAATCGGCTCC	plasmid construction for AGO3	2604
AGO3_R	GGGGATCCTTAAGCGAAGTACATTGT		
AGO4_F	GGTCCGGAGCCGCCATGGAGGCGCTGGGAC	plasmid construction for AGO4	2608
AGO4_R	GGGTCGACTCAGGCAAATAGATCGTGTG		
AGO2_F	GGGGATTCGCCACCATGTACTCGGGAG	plasmid construction for AGO2 wildtype	2599
AGO2_R	GGGGATTCAGCAAAGTACATGGTGCGCA		
AGO2_mutant_F	CGTGCGCGTGCAGCGGGACCGGCAGGAGATCA	plasmid construction for AGO2 mutant	7.3k
AGO2_mutant_R	TGATCTCCTGCCGGTCCCGCTGCACGCGCACG		
AGO1_qF	GAGGTCTGTAACATTGTGGC	quantitative PCR for AGO1 mRNA	130
AGO1_qR	TCTTCATCAGGCGACTGATC		
AGO2_qF	CCTGGAGGTCTGTAACATTG	quantitative PCR for AGO2 mRNA	166
AGO2_qR	ACGGACGTATGGATCTGTGT		
AGO3_qF	CAAGGCAACAGCAAGATCTG	quantitative PCR for AGO3 mRNA	138
AGO3_qR	CGTCCAGTTACATGAGCCAT		
AGO4_qF	GCAAGTATGCACAAGGTGCA	quantitative PCR for AGO4 mRNA	141
AGO4_qR	GGGTATCTCCAACACGTTTC		
CENPC1_qF	TCAGGATCATCTCAGAATAG	quantitative PCR for CENPC1 mRNA	140
CENPC1_qR	GCAGTTGCCGCATGCCTAAC		
18S_qF	CGGCGACGACCCATTCGAAC	quantitative PCR for 18S RNA	99
18S_qR	GAATCGAACCCTGATTCCCCGTC		
Dicer_qF	GGTGGTCCACGAGTCACAAT	quantitative PCR for Dicer mRNA	189
Dicer_qR	TGGTGGACCAACAATGGAGG		
α-sate_qF	CATTCTCAGAAACTTCTTTG	quantitative PCR for α-satellite RNA	120
α-sate_qR	AAGCGCTCCAAATATCCACT		
GAPDH_qF	CTTCATTGACCTCAACTACATGG	quantitative PCR for GAPDH mRNA	134
GAPDH_qR	CTCGCTCCTGGAAGATGGTGAT		
XIST_qF	TCAGCCTTCCCACCTGAAG	quantitative PCR for XIST RNA	151
XIST_qR	AGCAAGAGAAACATGGAAATGG		
LINE-1_F	TAATACGACTCACTATAGGGTGAGATCAAACTGCAAGGCG	in vitro transcription for Line 1 derived RNA	191
LINE-1_R	TGTCTGTGCCCTGCCCCCAG		
α-sate_F	TAATACGACTCACTATAGGGCATTCTCAGA AACTTCTTTG	in vitro transcription for α-satellite RNA	191
α-sate_R	CTTCTGTCTAGTTTTTATGT		
α-sate_tF	CTCAGAAACTTCTTTGTGAT	T-vector construction of α-satellite for Northern blot and FISH RNA probe	149
α-sate_tR	GTGAAGATATTTCCTTTTCC		
scramble siRNA	UUCUCCGAACGUGUCACGU	scramble siRNA control	N.A.
AGO2 siRNA_h_1	GGGUAAAGUUUACCAAAGA	AGO2 siRNA for human cells	N.A.
Dicer siRNA_h_1/m	GCCAAGGAAAUCAGCUAAA	Dicer siRNA for human cells and mouse cells	N.A.
AGO2 siRNA_h_2/m	GCCUGUAUCAAGCUAGAAA	AGO2 siRNA for human cells and mouse cells	N.A.
Dicer siRNA_h_2	GACGCUGAUUCAGCAUACU	Dicer siRNA for human cells	N.A.
CENPC1 siRNA_h_1	CCUUCAGGUAACUAUUAUA	CENPC1 siRNA for human cells	N.A.
CENPC1 siRNA_h_2	CCUCAGACCAGUGGAUAUA	CENPC1 siRNA for human cells	N.A.
ASAT siRNA_1	UUUUGAAACACUCUUUUUGU	exogenous ASAT siRNA	N.A.
ASAT siRNA_2	AGAAUCUGCAAGUGGAUAUU	exogenous ASAT siRNA	N.A.
